# Is it possible to attain the same soil organic matter content in arable agricultural soils as under natural vegetation?

**DOI:** 10.1177/00307270221082113

**Published:** 2022-02-21

**Authors:** David S. Powlson, Paul R. Poulton, Margaret J. Glendining, Andy J. Macdonald, Keith W.T. Goulding

**Affiliations:** 15552 Rothamsted Research, Harpenden, AL5 2JQ, UK

**Keywords:** Soil carbon, soil health, climate change, carbon sequestration, rewilding, arable cropping, natural vegetation

## Abstract

Clearing natural vegetation to establish arable agriculture (cropland) almost invariably causes a loss of soil organic carbon (SOC). Is it possible to restore soil that continues in arable agriculture to the pre-clearance SOC level through modified management practices? To address this question we reviewed evidence from long-term experiments at Rothamsted Research, UK, Bad Lauchstädt, Germany, Sanborn Field, USA and Brazil and both experiments and surveys of farmers’ fields in Ethiopia, Australia, Zimbabwe, UK and Chile. In most cases SOC content in soil under arable cropping was in the range 38–67% of pre-clearance values. Returning crop residues, adding manures or including periods of pasture within arable rotations increased this, often to 60–70% of initial values. Under tropical climatic conditions SOC loss after clearance was particularly rapid, e.g. a loss of >50% in less than 10 years in smallholder farmers’ fields in Zimbabwe. If larger yielding crops were grown, using fertilizers, and maize stover returned instead of being grazed by cattle, the loss was reduced. An important exception to the general trend of SOC loss after clearance was clearing Cerrado vegetation on highly weathered acidic soils in Brazil and conversion to cropping with maize and soybean. Other exceptions were unrealistically large annual applications of manure and including long periods of pasture in a highly SOC-retentive volcanic soil. Also, introducing irrigated agriculture in a low rainfall region can increase SOC beyond the natural value due to increased plant biomass production. For reasons of sustainability and soil health it is important to maintain SOC as high as practically possible in arable soils, but we conclude that in the vast majority of situations it is unrealistic to expect to maintain pre-clearance values. To maintain global SOC stocks at we consider it is more important to reduce current rates of land clearance and sustainably produce necessary food on existing agricultural land.

## Introduction

One aspect of “biomimicry” as applied to agriculture is the idea of maintaining soil in as near as possible the same state as it was under natural vegetation. A specific example of this is the goal of maintaining, or restoring, soil organic matter (SOM) to the content that existed before a site was cleared of natural vegetation. This goal is often implied rather than stated explicitly. A recent example is inclusion of an estimate of soil carbon loss compared to an assumed value under “natural” conditions in the “debt based approach to land degradation as an indicator of global change” proposed by Wuepper et al. (2021). It also sometimes implied in discussions of soil health or quality or topics such as ‘regenerative agriculture’ and ‘carbon farming’.

A driver for interest in the topic is the suggestion that sequestering additional organic carbon in soils can make a significant contribution to mitigating climate change. This was encapsulated in a poster at a protest for climate justice in Berlin in 2021, ahead of the COP 26 conference in Glasgow, stating “CO_2_ is in the air, let's bring it back to our soils!” However, there is vigorous debate concerning the extent to which this can be achieved (e.g. Minasny et al., 2017; Poulton et al., 2018; Schlesinger and Amundson, 2019; VandenBygaart, 2018). Irrespective of this debate, the benefits of increased SOM for soil health and functioning are well recognized (Allison, 1973; Janzen, 2015).

Soils growing annual crops are treated in several obviously different ways to those under natural vegetation, suggesting intuitively that it will be difficult to maintain the same content of organic matter. These differences include the following:

1. Much of the plant biomass is removed because the goal of agriculture is to produce products (food, fibre, fuel) for human use elsewhere. Under natural vegetation the majority of plant material remains on the site and enters the soil. The exceptions are that removed by herbivory, very small quantities removed by humans, typically fruits or nuts harvested by indigenous people, and biomass destroyed by fire in situations where this occurs naturally. Within agricultural systems, biomass removal can be partially counteracted by decisions regarding the proportion of plant material that is returned to the soil, e.g. in crop residues such as straw or as manure after being processed by animals or humans. Removal of biomass also exports nutrients from the soil which, if not replaced, will limit the capacity for future biomass growth and return of organic residues.
2. New plants are established from seed (or transplanted seedlings in the case of flooded rice) each year in contrast to the dominance of perennial plants in natural vegetation that can build up extremely large root systems over periods of years.
3. The soil is often mechanically disturbed once or more each year through tillage and planting operations. Zero till methods can be adopted to avoid this, at least partially.

Where natural vegetation is converted to agricultural pasture some of these differences do not apply, or are less pronounced (Murty et al., 2002; Guo and Gifford, 2002). In this article we focus on arable agricultural systems and do not discuss the ways that different grazing managements may positively influence the build-up of organic carbon. We only include consideration of pasture in its context as part of mixed arable/grassland agricultural systems, sometimes termed ley-arable.

## Goal of this article, limitations and definitions

In this article we attempt to compare the organic carbon content of soil under a range of arable cropping practices with that in soil at the same site prior to clearance of natural vegetation. We use data from long-term experiments, chronosequences and surveys of soils under different land uses. As we will see, simple comparative data is in short supply and, in many cases, proxies and deductions are necessary to estimate the likely pre-clearance SOC content. With a very few exceptions, comparing SOC in cropped soil with that under natural vegetation was not the main objective of the studies: often the aim was to assess crop yield trends, with measurements on soil being an extra. For this reason, combined with the limited amount of available data and the different ways in which SOC values are reported (concentration or stock, different depths; see below), meant that a formal and quantitative meta-analysis based on a literature search using specified criteria was not feasible. Consequently we do not attempt a comprehensive review, but rather present a series of case studies which we consider representative of many situations. The examples include temperate, sub-tropical and tropical environments in five continents.

Soil organic matter (SOM) contains about 50% carbon, C (Pribyl, 2010), referred to as soil organic carbon (SOC). We use both abbreviations in this article. We only consider organic C but recognize that some soils contain significant quantities of inorganic C as carbonates; these must be taken into account when analysing soils and carbonate can be lost as a result of some agricultural practices. There is also evidence that addition of basaltic rock to agricultural soils may sequester C in inorganic forms, potentially contributing to climate change mitigation (Kelland et al., 2020), but we do not address soil inorganic C transformations in this article.

When discussing SOC changes over time it is preferable to compare SOC *stocks*, typically in units such as t C ha^−1^ rather than SOC *concentration* in units such as %C or mg C kg^−1^ soil (Murty et al., 2002; Don et al., 2011), and where possible we do this. But to obtain stock values it is necessary have a value for soil bulk density or soil mass to a defined depth and this is often not measured and it is common for authors to only quote SOC concentration; in reviewing data from published literature we have had to use the data as published. On the basis of their review of land use change impacts in tropical regions, Don et al. (2011) concluded that failure to use stock values, as influenced by changes in soil bulk density, led to underestimating SOC changes by 28%.

## Setting the scene – loss of soil organic carbon after clearing natural vegetation

**Figure 1.**
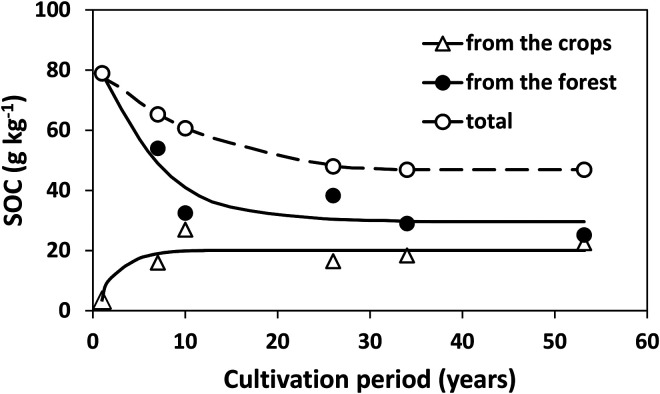
Changes in soil organic carbon following clearing of forest in Ethiopia. Redrawn from Lemenih et al. (2005).

Figure 1 shows an example of deforestation causing a loss of SOC in Ethiopia, based on a chronosequence of cleared sites used for growing maize and sorghum compared with an area of remnant forest (Lemenih et al., 2005). Within 25 years SOC in the surface 10 cm had decreased by 42%, mainly through decomposition though some was translocated to the 10–20 cm layer due to tillage. This major loss of C was despite the soil being of volcanic origin, and thus having a greater capacity to retain organic matter than most other soil types because of the properties of clay minerals present. Because the introduced crops had the C-4 photosynthetic pathway, C derived from them could be distinguished from that derived from the original C-3 forest vegetation using ^13^C isotopic measurements. More than half of the forest-derived C was lost within 35 years but that remaining after this was relatively stable. After 53 years >40% of the SOC present was derived from the introduced crops but there was evidence that this “new” C had a faster turnover rate than C remaining from the forest. Losses of SOC after clearing natural vegetation and conversion to arable cropping have also been shown in several earlier reviews including Murty et al. (2002), Guo and Gifford (2002) and Don et al. (2011).

**Figure 4.**
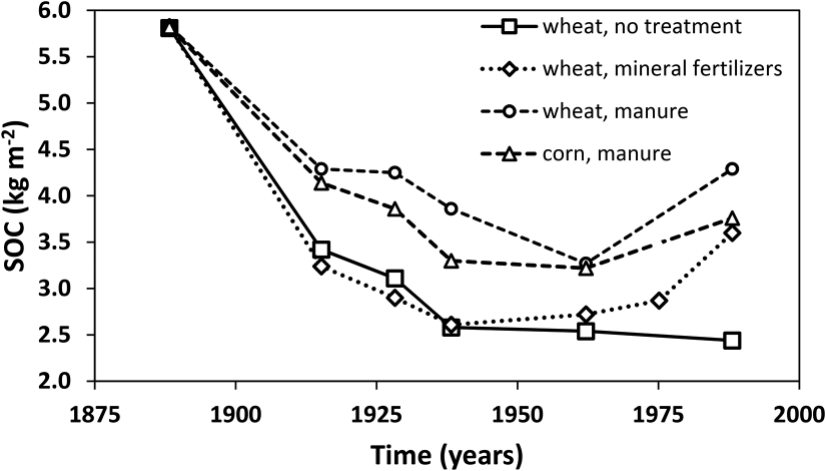
Soil organic carbon in selected treatments of the Sanborn Field long-term experiment, University of Missouri-Columbia, USA. Redrawn from Buyanovsky and Wagner (1998).

**Figure 5.**
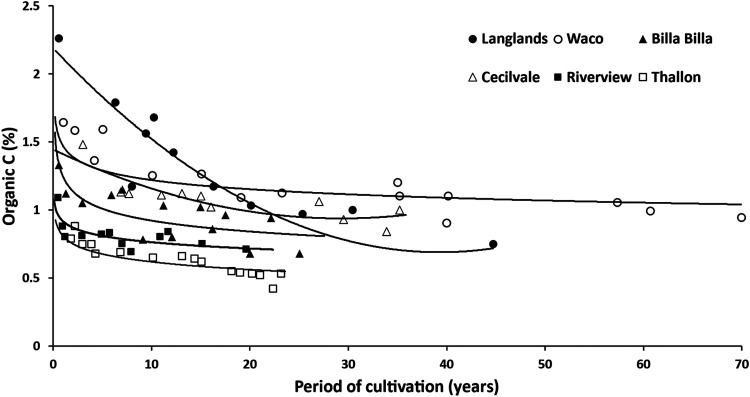
Changes in soil organic C (0–10cm depth) after clearing natural vegetation and converting to arable crops at 83 sites on 6 soil series in a sub-tropical region of southern Queensland, Australia. Redrawn from Dalal and Mayer (1986). See Table 4 for information on soil properties and management at sites.

**Figure 6.**
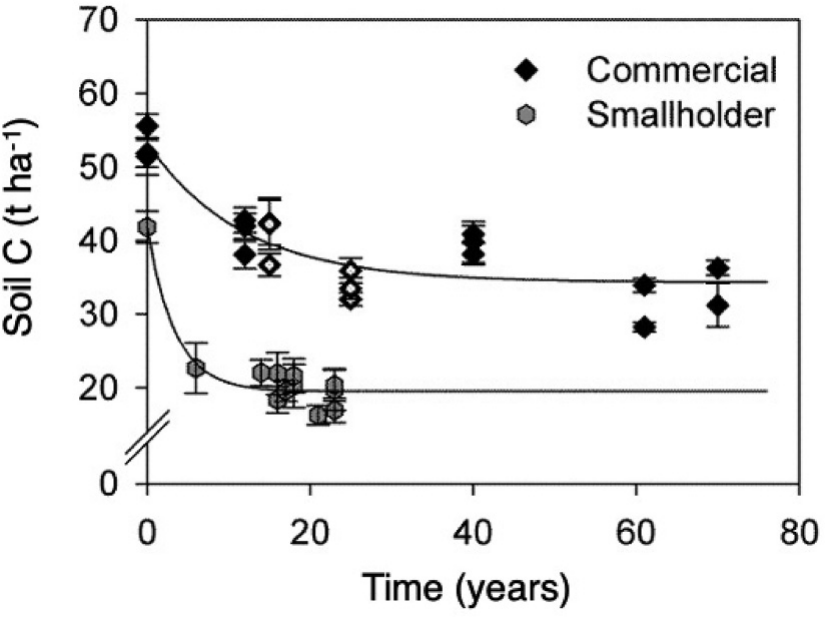
Changes in soil organic carbon following clearance of natural vegetation in Zimbabwe. Redrawn from Zingore *et al*. (2005).

Figure 1 illustrates a central principal in our understanding of SOC dynamics: following a change in management SOC content tends to move asymptotically towards a new equilibrium value and neither increase nor decrease continually. This can also be seen following land clearance at Sanborn Field, USA (Figure 4), and examples from Australia and Zimbabwe (Figures 5 and 6) discussed later. The trend is also seen in data from the long-term experiments at Rothamsted where manure is applied annually or there is a change in land use (Macdonald, 2018; Macdonald et al., 2017; Johnston et al., 2009; Poulton et al., 2018). In farming practice, as opposed to experiments, such equilibrium values (sometimes termed steady state or quasi-steady state) may never be actually observed because soils are subject to many different management changes over time, some causing an increase in SOC and some a decrease, with these being superimposed on each other.

## Long-term experiments at Rothamsted and Woburn experimental farms, UK

### Establishing a proxy value for soil organic carbon under natural vegetation at Rothamsted

At Rothamsted, as for many very long-running experimental sites, specific measurements of pre-clearance SOC do not exist so it is necessary to estimate a proxy value indirectly. It is known that the natural forest that covered much of the UK was initially cleared in south-east England in pre-Roman times, over 2000 years ago, and the remains of a Roman Mausoleum (Scheduled Monument; List Entry Number: 1018377) can be found on the farm. Although it is likely that specific areas may have moved into and out of cropping since then, a map of 1623 shows several fields, including Broadbalk and Geescroft, as arable at that time. There are written records of arable cropping on these fields from the 1840s, with the Broadbalk Wheat Experiment starting in 1843. Sections of Broadbalk and Geescroft fields were left unharvested from 1882 and 1886, respectively, and have reverted to deciduous woodland, a process now termed passive rewilding (Broughton et al., 2021); see Poulton et al. (2003) for details. We suggest that the SOC stocks in these sites, following some 120 years of woodland reversion, may be a reasonable indication of the SOC before initial clearance. Table 1 shows the SOC at both sites when measured in 1999. The Broadbalk wooded area contained 78 t C ha^−1^ in the 0–23 cm depth (3.45% C) and a total of 123 t C ha-^1^ to a depth of 69 cm; the corresponding values for Geescroft were 63 t C ha^−1^ (2.61% C) and 105 t C ha^−1^ and both sites contained additional organic C in tree roots. The lower SOC content of Geescroft reflects the decline in soil pH at this site, as discussed by Poulton et al. (2003). It is likely that the SOC stocks at both sites are an underestimate of the initial pre-clearance value because above-ground tree biomass was still increasing at the time of the 1999 measurements so inputs of organic C to soil were probably continuing to increase. More generally, there is evidence that SOC in afforested areas often does not reach that under a comparable natural forest (Laganière et al., 2010). We therefore take the SOC content of the Broadbalk Wilderness site as a conservative estimate of pre-clearance SOC: 80 t C ha^��1^ or 3.5% C, both rounded to avoid a spurious appearance of accuracy.

**Table 1.** Estimations of soil organic carbon in soil at Rothamsted before clearance of natural woodland based on sites under natural regeneration since 1882 (Broadbalk Wilderness) or 1886 (Geescroft Wilderness). From Poulton et al. (2003).

Site	Organic C concentration in 0–23 cm depth soil %	Organic C stock, t ha^−1^		
		0–23 cm	0–69 cm	Roots*
Geescroft Wilderness	2.61	63**	105**	45
Broadbalk Wilderness wooded section	3.45	78	123	75
Broadbalk Wilderness grassland section	3.10	72	113	-

* Estimated from tree above-ground biomass but refers to a soil depth of 0–69 cm; see Poulton et al. (2003).

** Including surface litter. Only present in significant quantity at Geescroft Wilderness due to low soil pH.

### Impacts of management practices on soil organic carbon

Table 2 shows the SOC content of soils from several long-running experiments at Rothamsted Farm. For all soils that had been in long-term arable cropping (22–167 years) with typical rates of fertilizer and other management practices, but no special measures to increase SOM content, SOC was around half (31–53%) of that in Broadbalk Wilderness, taking this as a proxy for the pre-clearance value. There are some small but interesting differences between these treatments. The continuous wheat on Broadbalk receiving 144 kg N ha^−1^ annually, an N fertilizer application rate close to the current UK average for arable crops (137 kg N ha^−1^; Defra, 2020) contained more SOC in the plough layer soil (0–23 cm) than the unfertilized plot (38 cf. 31 t C ha^−1^). Organic C inputs from roots, root exudates and stubble are slightly increased where yield is increased by N application (Jenkinson et al., 1992). The trend for long-continued N fertilizer applications to lead to increased SOM content through this mechanism was discussed in detail for the Broadbalk Experiment by Glendining et al. (1996) and reviewed for long-term experiments globally by Glendining and Powlson (1995) and Ladha et al. (2011).

**Table 2.** Soil organic carbon in selected contrasting treatments in long-running experiments at Rothamsted Research, UK.

Experiment name	Long-term land use	Year experiment started	Period of treatments,years	Treatment or land use in experiment	Organic C in soil (0–23 cm or equivalent sampling depth) at end of treatment period	C content compared to Broadbalk Wilderness %	Data source
					t C ha^−1^		
Broadbalk	Arable	1843	167	Winter wheat, no inputs	24.5	31	Powlson et al. (2012)
				Winter wheat, N_3_PK annually	30.0	38	
				Winter wheat, FYM annually	78.4	98	
Fosters Ley-arable	Arable	1949	59	Continuous arable crops	37.9	47	Poulton et al. (2018)
				Ley-arable rotations: 3yr ley, 3yr arable*	50.2	63	
				Continuous grass or grass/clover (seeded at start of experiment)	63.5	79	
Highfield Ley-arable	Grass since 1838	1949	59	Continuous arable crops	36.1	45	Poulton et al. (2018)
				Ley-arable rotations: 3yr ley, 3yr arable*	49.4	62	
				Continuation of original grass	73.2	92	
Rates of straw	Arable	1987	22	Straw removed	53.2	67	Poulton et al. (2018)
				Straw (rate 1)**	56.1	70	
				Straw (rate 2)	59.2	74	
				Straw (rate 4)	61.2	77	

* 3year ley was grazed grass 1949–1962. Changed to cut grass/clover mixture in 1962.

** Rate 1 represents return of the amount of straw grown on the site. Rates 2 and 4 are 2 and 4 times this quantity, obtained by importing straw.

The only treatment in the Broadbalk Experiment to increase SOC in continuous arable soil to a level similar to that found in woodland was the annual application of farmyard manure (FYM), but the very high annual application rate of 35 t ha^−1^ (containing approx. 220 kg N ha^−1^) means that this must be regarded as an extreme experimental treatment, not a practical recommendation. This quantity of manure would rarely be available in practical situations and, even if it was, applying it annually leads to serious water pollution by nitrate and phosphate, as measured in drainage from the Broadbalk plots (Goulding et al., 2000; Heckrath et al., 1995). Consequently, manure applications are limited to 170 kg N ha^−1^ annually within Nitrate Vulnerable Zones in the EU and equivalent areas elsewhere.

The Highfield and Fosters Ley-arable experiments are both located within 1km of Broadbalk and are on the same soil type, so it is reasonable to assume the same pre-clearance SOC value. Both started in 1949 but Highfield had been in grass since 1838 and initially had a much higher SOC content than Fosters that had been arable for many years. However, after 59 years of continuous arable cropping much of the legacy of higher SOC in Highfield had been lost and both had similar SOC contents (Table 2). At both sites the ley-arable rotation (3 years arable, 3 years pasture) led to increased SOC compared to continuous arable, but only achieved about 60% of the presumed pre-clearance value. It was only the continuous pasture treatments that achieved an SOC approaching a pre-clearance value: 79% in Fosters and 92% in Highfield.

Annual application of cereal straw at the rate grown on that soil in the “Rates of straw” experiment on Rothamsted Farm (rate 1 in Table 2) had only increased SOC slightly after 22 years. It was only when double or quadruple the normal rate was applied, by importing straw from elsewhere, that the increase became statistically significant, increasing to 60% of the presumed preclearance value. Obviously, such high rates of straw could not be applied to all arable land.

The SOC values in Table 2 are consistent with a survey of over 50 fields on Rothamsted Farm that are not in long-term experiments (Johnston et al., 1981). At the time the fields were sampled, those with a mainly arable history, or with only short periods (< 3 years) under grass in the previous 20 years, had SOC in the range 1.2–1.7%, similar to the values of 1.0–1.5% in the long-term arable treatments in Table 2. A group of fields under arable but with longer leys had a mean SOC of 1.6%. It was only those fields that had been in grass for long periods that had SOC around the presumed pre-clearance value of 3.5%.

**Figure 2.**
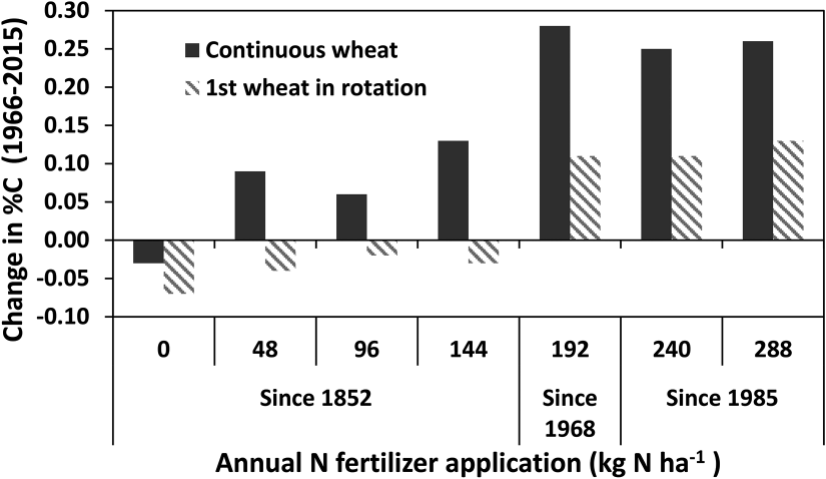
Changes in SOC concentration (expressed as %C) in 0–23 cm depth in selected treatments of the Broadbalk Wheat Experiment at Rothamsted Research, UK, between 1966 and 2015. Dark bars – winter wheat grown continuously every year. Hatched bars bars – wheat grown in rotation. See Glendining et al. (2021) for details of crop rotations.

Growing arable crops in a rotation, instead of the same crop being grown continuously for successive years, delivers many agronomic benefits, a major one being decreased incidence of pests and diseases compared to monocultures. This is demonstrated in the Broadbalk Experiment by the higher yields of winter wheat grown in a rotation compared to continuously (Macdonald et al., 2017). In addition, crop rotations almost certainly lead to increased biodiversity of plant and insect life in and around fields. But do they lead to increased SOC? Two sets of data from Rothamsted experiments indicate that, at best, any positive effect is small. Figure 2 shows changes in SOC in the 0–23 cm depth of selected treatments of the Broadbalk Experiment receiving inorganic fertilizers, but expressed as changes in %C rather than C stock. Until 1968 winter wheat was grown every year in all treatments of the experiment, apart from fallowing in some years to control weeds, but in 1968 a crop rotation was introduced within certain sections of each fertilizer treatment. The SOC changes in Figure 2 refer to the 49 year period from 1966 to 2015 during which continuous wheat and wheat in rotation can be compared. The rotation has changed at different times but crops have included potatoes, forage maize and fallow years; see Glendining et al. (2021) for details. For continuous wheat SOC generally increased with increasing N fertilizer application up to the 192 kg N ha^−1^ rate (Figure 2), consistent with the data in Table 2 and as discussed by Glendining et al. (1996). For soils under the crop rotation there were small decreases in SOC for the lower N rates; at the three highest N rates there were increases but smaller than under continuous wheat. In part these results reflect decreased plant inputs under rotation due to the fallow years, and so are not representative of commercial practice but there will also be decreased inputs from the spring-sown crops as the soil is bare for much longer periods than under winter wheat and the trend in commercial practice is to include more spring-sown crops where rotations are introduced.

**Figure 3.**
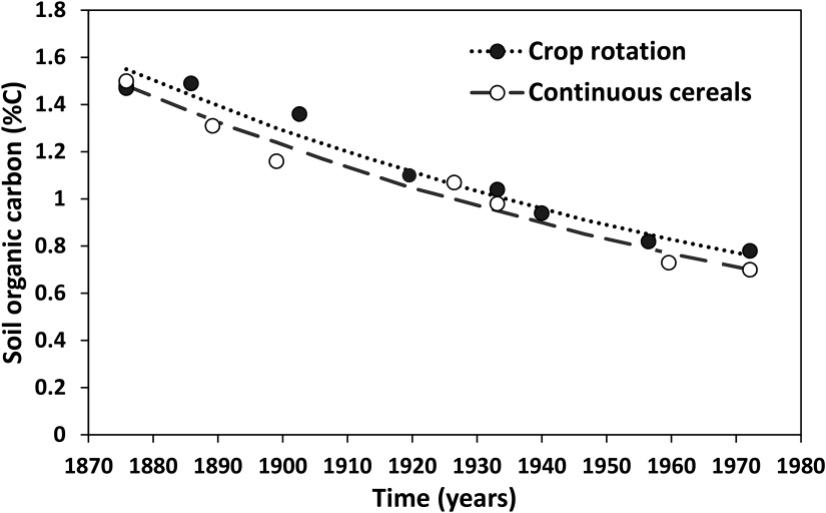
Comparison of soil organic carbon (%C) in 0–23 cm depth in soil growing cereals (wheat and barley) continuously and that in a crop rotation in Stackyard Field, Woburn Experimental Farm, UK, between 1876 and 1974. Adapted from Mattingly et al. (1975).

Figure 3 shows a somewhat different trend from a comparison of continuous cereals (wheat and barley) and a crop rotation on a sandy soil at Woburn Experimental Farm some 50 km from Rothamsted. The soil here contains 10–15% clay in contrast to around 25% clay at Rothamsted. The data shown (from Mattingly et al., 1975) is from treatments receiving only inorganic fertilizers, with no manure applications. These measurements made over almost 100 years show a small positive effect of crop rotation. Several factors probably contributed. In some years there was crop failure in the continuous wheat or barley treatments due to disease, so the soil was effectively bare, and the rotation treatment included a one-year grass ley at various times. This trend towards slightly increased SOC, whilst beneficial for soil health, would certainly be insufficient to restore the soil to a pre-clearance level. The decrease in SOC apparent in both treatments during the 20^th^ century is probably due to a continuing slow decline from a previous higher value as it is known that the site was under grass earlier in the 19^th^ century, before the experiment started.

Numerous experiments have been conducted at the Woburn site including many measurements of SOC under different management practices. But estimates of pre-clearance SOC content at this site are even more uncertain than at Rothamsted. One indication is a measurement of 1.5% C in topsoil at Stackyard field in a sample taken in 1876 at the beginning of a series of experiments in that field that continued into the 20^th^ century (the initial value shown in Figure 3). Records show that this field had been in grass for 50 years until the 1820s, then converted to arable cropping. We speculate that, when under grass, SOC would have been about 2% C or slightly higher which compares with a typical range of 0.6–0.9% C currently found in arable fields on this soil type at Woburn Farm. The assumed pre-clearance value is roughly equivalent to 70 Mg C ha^−1^ in topsoil taking a soil bulk density value for the soil type from Johnston et al. (2017).

Table 3 shows changes in SOC over the period 1936 to 2009 in the Woburn Ley-Arable Experiment, a study similar to the ley-arable experiments on the silty clay loam soil at Rothamsted Experimental Farm (Table 2). The experiment is complex, with various changes to treatments over time; Table 3 shows a simplified summary of the contrasting crop rotations but see Johnston et al. (2017) for full details. In the all-arable rotation SOC in the plough layer soil remained in the range 36–39 Mg C ha^−1^ throughout the 70 year period, but slightly less where the rotation included root crops or fallow years. With ley-arable rotations comprising 3 years ley followed by 2 years of arable crops, SOC increased but the maximum values achieved were 46–48 Mg ha^−1^, well below the presumed pre-clearance value of about 70 Mg ha^−1^. In the treatment with a 3 year grass/clover ley, lucerne had been grown for the first 34 years and this only led to a small increase in SOC. It increased markedly after the change to grass/clover from 1965. This is thought to be because lucerne has a deep tap-root as opposed to the mat of fine roots found beneath grass/clover. Thus, the organic C inputs from lucerne, at least into topsoil, are almost certainly less than from grass/clover emphasizing the importance of such details when considering ways of increase SOC. From 35 years since the start of the experiment rotations comprising 8-year leys followed by 2 years of arable cropping were introduced. As expected, these caused a further increase in SOC compared to the 3-year leys, reaching >50 Mg ha^−1^. If they had continued for the whole 70 year period it is possible SOC in these treatments would have reached values close to that presumed under natural vegetation of about 70 Mg ha^−1^. However, this rotation would be predominantly pasture with only 2 arable crops every 10 years. If applied commercially it would necessarily require livestock to utilize the pasture and the agricultural system would become mainly animal based.

**Table 3.** Changes in soil organic carbon content as influenced by different crop rotations in the Woburn Ley Arable Experiment. Crop rotations shown are a simplified summary; see Johnston et al. (2017) for full details including variations in treatments over time. Data refers to the 0–25 cm soil depth, though adjusted according to changes in soil bulk density to give “equivalent sampling depth”, i.e. equal mass of soil sampled in each treatment. During the early years of the experiment leys were grazed, but later this changed to cutting.

Crop rotation	Soil organic carbon in 0–25 cm depth, Mg C ha^−1^		
	1938	1965–1974^a^	2000–2009^a^
All arable	36.9	38.7	35.5
All arable including fallows and root crops	36.9	35.2	31.0
3yr ley (grass + N), 2yr arable	36.9	47.1	47.6
3 yr ley (grass/clover), 2yr arable^b^	36.9	40.3	45.9
8 yr ley (grass + N), 2 yr arable	36.9	39.7	52.3
8 yr ley (grass/clover), 2 yr arable	36.9	41.4	51.3

^a^ Range of years refers to different times when each rotation was phased into the experiment.

^b^ Initially ley was lucerne, but in the 1965–1974 period was changed to grass/clover mixture.

## Long-term experiments globally

The Bad Lauchstädt Static Fertilization Experiment in Germany started in 1902 and has similarities to the Broadbalk Experiment at Rothamsted. It comprises an all-arable crop rotation which at various times has included winter wheat, winter and spring-barley, sugar beet, maize and potatoes. These receive either manure (FYM), inorganic fertilizers, or both. SOC in treatments receiving only inorganic fertilizers has remained at about 1.8% C for many years (Körschens, 2021). With FYM applied at 20 or 30 t ha^−1^ in alternate years, SOC reached 1.9 and 2.2%C, respectively. These rates of manure are high, though less unrealistic than in the Broadbalk experiment. However, they are probably outside the limits permitted in Nitrate Vulnerable Zones in view of concerns about N and P movement to natural waters. The Bad Lauchstädt experiment is on a chernozem soil, developed under steppe vegetation comprising natural grassland and some trees (Altermann et al., 2005). As for Rothamsted, no SOC value is available for the soil under natural conditions and this is further complicated at this site because there is evidence of farming in the region from Neolithic times. In 1988 a natural regeneration treatment was introduced; in this SOC increased from 2.1% C to 3.0% C over 32 years (Ines Merbach, personal communication) and it may be that this is moving towards some indication of a “natural” SOC content. SOC in the arable treatments of the experiment is well below this: 60% of this value in plots receiving only inorganic fertilizers and 73% with the highest rate of manure.

Ladha et al. (2011) reviewed SOC data from 114 long-term experiments globally. The aim of the review was to assess the impact of inorganic N fertilizer on SOC and it was concluded that in the vast majority of cases N application led to small increase in SOC compared with treatments not receiving N, as found in the Broadbalk experiment (Table 2 and Powlson et al., 2010). However, a significant observation was that in many LTEs soil C was declining in all treatments, whether given N fertilizer or not; in these cases, the effect of N application was to slow the decline rather than cause an absolute increase. The sites where SOC was declining across all treatments were generally where natural vegetation had been cleared about 100 years or less before the start of the experiment, typically natural grassland prairies in North America. This was in contrast to the very oldest experiments in Europe such as Broadbalk and Bad Lauchstädt where SOC appears to have declined to a new equilibrium level.

Martel and Paul (1974) give examples of prairie clearance at sites in Canada on chernozem soils that typically accumulate a high SOC content during soil formation. At a site where the soil contained 25% clay, SOC had declined to 65% of the original value after 15 years of arable cropping and to 41% after 60 years, based on sampling of the 0–10 cm soil depth. At similar sites the declines were to 47–68% after 30 years. At a site with a much larger clay content (70%) the decline was to 81% of the original value after 20 years of cropping, reflecting the greater capacity of high-clay soils to stabilize and retain organic C.

The Sanborn Field Experiment on a silt loam soil at the University of Missouri-Columbia was established in 1888. Handwritten notes describe the original Prairie vegetation as “grass with scattered woody species” (Buyanovsky et al., 1996). Figure 4, adapted from Buyanovsky and Wagner (1998), shows changes in SOC for selected treatments. After 100 years SOC in the treatment growing winter wheat continuously with inorganic fertilizers had declined to 62% of the starting value; where manure was applied it was 74%. In reality the decreases may be even greater as it is not entirely clear whether the initial SOC value shown is from beneath the native vegetation or measured soon after initial ploughing. From about 1950 new higher-yielding crop varieties were introduced and above-ground residues returned to the soil (Buyanovsky and Wagner, 1998) and these changes led to clearly measurable increases in SOC in most treatments, but still far below the initial value under prairie (Figure 4). It is reported that SOC was also greater in a treatment where timothy grass (*Phleum pratense* L.) was grown instead of annual crops (Buyanovsky et al., 1996).

## Surveys of different land uses in specific regions

### Australia

Dalal and Mayer (1986) reported SOC changes in soils of the so-called cereal belt of southern Queensland, Australia, using samples taken from 83 farmers’ fields that had been cultivated, after clearance of natural vegetation, for periods ranging from 0.5 to 70 years. The region is sub-topical with mean annual temperatures in the range 18.5–20.5°C and mean annual rainfall 480–670 mm. The natural vegetation was predominantly trees of the species *Acacia*, *Eucalyptus* and *Casuarina* and the perennial grass *Dichanthium sericeum*. The main crops grown after clearance were wheat, barley, sorghum and sunflower. Generally, two crops per year were grown but cropping was often interspersed with 6–12 month periods of fallow, a practice common in the region due to low and variable rainfall. Nitrogen fertilizer applications were low, ranging from zero to a maximum of 60 kg N ha^−1^ per crop. 

Figure 5 shows the decline in SOC concentration in the 0–10 cm depth (expressed as % C), with the sites grouped according to the 6 soil series represented, and the mean data as fitted by a simple decomposition model that assumed SOC moved to a new equilibrium value. Further information about the sites is shown in Table 4 together with SOC stocks in cultivated soils as a percentage of pre-clearance values. At all sites there were large losses of SOC after clearance. The sharpest decline (66% in soil C concentration within one year; Figure 5) was in the soil with the lowest clay content (18% clay, compared to 34–72% at the other sites); these sites also had the least proportion of straw returned to the soil: only 14% of crops compared to >50% at most other sites (Table 4). 

Averaged over sites within each soil series, and over the different periods after clearance, SOC stock had declined to 35–61% of pre-clearance values (Table 4). The absolute decreases were least in the soil type with the smallest pre-clearance value (associated with low rainfall in this region leading to low biomass of natural vegetation) and greatest at the sites starting with the highest values. Management factors such as frequency of fallowing, extent of crop residue return and frequency and type of tillage operations will have influenced the rates of SOC loss but their relative impacts cannot be quantified from the published data because they are confounded with the number of years since clearance. SOC losses were also measured in soil to a depth of 30 cm at some sites, but were proportionately somewhat less than in the 0–10 cm depth.

### Zimbabwe

Zingore et al. (2005) reported SOC losses following clearance of natural or semi-natural vegetation in Zimbabwe and conversion to maize-based cropping. Figure 6 shows data for clay soil sites (Chromic Luvisols, containing 32–35% clay, under tropical conditions; mean annual temperature 24°C and mean annual rainfall 800–1000 mm). The cleared vegetation was dense woodland comprising *Brachystegia spiciformis* and *Julbernardia globiflora* with a grass understorey dominated by *Hyparrhenia* spp. Results showed a marked contrast between the loss of SOC between smallholder management and commercial farms. Under smallholder conditions SOC declined to 47% of the pre-clearance value within 25 years, with much of this decline occurring within 3 years. Under commercial farm management the decline was much slower, reaching 64% of the pre-clearance value in 25 years and stabilizing at a higher SOC content of around 35 t C ha^−1^ compared to < 20 t C ha^−1^ under smallholder conditions. In the situation described by Zingore et al. (2005) smallholders applied little or no fertilizer to crops, generally achieving maize yields of <2 t ha^−1^, and maize stover was mostly grazed by cattle. By contrast, on commercial farms, maize typically received 160–200 kg N and 3–35 kg P ha^−1^, giving grain yields of 7–8 t ha^−1^ except in years when limited by drought. Crops produced 10–12 t stover ha^−1^, of which at least 80% was returned to the soil. The contrast in the two management practices emphasizes the importance of organic inputs to soil, both via the roots of larger crops and from crop residues. It also demonstrates the influence of socio-economic factors, especially for smallholder farmers, with the competition for use of crop residues for soil improvement or animal fodder.

### United Kingdom

The UK Countryside Survey reports data on soils under different land uses based on a sampling scheme comprising 591 1km x 1km samples squares. In 2007 SOC stock was, as expected, least in soils classed as arable and horticultural: 43.0 t C ha^−1^ in the 0–15 cm depth compared to 61.0, 62.4 and 66.3 t C ha^−1^ in the categories of improved grasslands, natural grasslands and broadleaved woodlands, respectively (Carey et al., 2008).

## Agroforestry

Mayer et al. (2022) reviewed data on sequestration of C under agroforestry systems at 61 sites in temperate regions. The sites ranged from 4 to 83 years since establishment and covered three main soil types, reflecting the main classes used for agriculture in temperate regions: Luvisols, Chernozems and Cambisols. Obtaining representative soil samples from agroforestry sites is challenging because SOC can be very different in the cropped areas compared to areas close to trees. The review showed increased SOC in the 0–20 cm depth in areas not immediately adjacent to trees in 72% of observations and in 81% in the 20–40 cm depth. The mean rates of C accumulation were 0.21 ± 0.79 and 0.15 ± 0.26 t C ha^−1^ yr^−1^ in these two depths, respectively. Whilst there are often many benefits from introducing agroforestry, there is no suggestion from this review that SOC can be increased to anything approaching that under natural, non-agricultural, land-use across the entire area. In an earlier review, Laganière et al. (2010) found that afforestation of croplands generally increased SOC of arable lands but not significantly in grasslands, presumably because these soils were already close to saturation.

## Some interesting exceptions

All of the examples given above from temperate (Europe, North America), tropical (Ethiopia, Zimbabwe) and sub-tropical regions (Australia) consistently show a substantial loss of SOC following clearance of natural vegetation and no indication that the original content can be restored through realistic management changes if the soil remains in mainly arable cropping. The examples given in this section show a contrast to this trend.

### Irrigated agriculture

In regions with low rainfall the introduction of irrigated agriculture increases annual plant biomass production. Even accounting for harvested material removed from the field, organic inputs to soil can be greater than under natural conditions leading to an increase in SOC; an example is the area of California with a Mediterranean or semi-arid climate. Increased SOC compared to that under natural conditions, following expansion of irrigation and related intensification of cropping, is illustrated by a comparison of soil properties in archived samples taken at 125 sites in the 1950s or earlier and samples taken at the same sites some 50–60 years later (DeClerck and Singer, 2003). During this period there was considerable intensification of agriculture in California based largely on expansion of irrigation. Averaged over all sites, SOC increased from 1.05 to 1.35%; in some regions the increase was considerably greater, e.g. from 0.9 to 1.5% in the Upper San Joaquin Valley. The San Joaquin Valley is the location of the long-term Century Experiment, established in 1993 by the University of California, Davis. The area was originally under oak savannah and perennial grassland but has been farmed with mostly row crops for at least 100 years (Wolf et al., 2018). SOC in many plots not treated with manures was at around 1% in the 0–15 cm depth when sampled in 2012 (Wolf et al., 2018). Within the experiment it has been found that cover crops and zero tillage caused substantial increases in SOC; for example, an increase from cover crops of 3.5 t C ha^−1^ in the 0–15 cm soil depth after 20 years (Mitchell et al., 2017).

### Long leys and volcanic soils

Table 5 (from Zagal et al., 2013) shows changes in SOC at two sites on volcanic soils in Chile after clearing natural grassland and conversion to various crop rotations. At one site, Diguillin, measurements were made 8 years after clearance and at the other, Santa Barbara, after 16 years. It is widely reported that the clay minerals present in volcanic soils, especially allophane which has a spherical structure, have a strong tendency to stabilize organic matter through a range of mechanisms and this can lead to large SOM contents accumulating in such soils (Parfitt, 2009; Buurman et al., 2007). This was especially so at Santa Barbara where 50% of the clay was allophane. At Diguillin SOC had declined to 71% of the pre-clearance value after 8 years of continuous arable cropping. In a 4-year rotation, comprising 2 years arable and 2 years red clover (repeated twice), the decline was less (Table 5). But replacing red clover with alfalfa failed to decrease the decline compared to continuous arable cropping – a similar finding to the effect of alfalfa in the Woburn Ley-arable experiment in UK (Table 3). Both 8 year rotations that included 5 years of either white clover or alfalfa kept the SOC content to 83 and 80%, respectively, of the original value under natural grassland. Even under continuous arable cropping, the losses at this site are less than for comparable periods in the Australian examples, presumably a combined result of the volcanic soils and cooler climate at the Chilean site.

**Table 4.** Changes in soil organic C following clearance of natural vegetation and arable cropping for various periods at 83 sites on 6 soil series in a sub-tropical region of southern Queensland, Australia. Adapted from Dalal and Mayer (1986).

Soil series	Clay content, %	Number of cultivated sites	Mean period of cultivation, years*	Percentage of crops where residues returned to soil %	SOC stock in soil under natural vegetation (0–10 cm depth), Mg C ha^−1^	SOC stock in cultivated soils (0–10 cm depth**), Mg C ha^−1^	SOC in cultivated soils compared to pre-clearance value, %
Waco	72	16	26.2 (1–70)	49	13.7 ± 0.22	8.3 ± 0.34	60
Langlands	49	12	18.0 (0.5–45)	77	22.1 ± 0.84	7.8 ± 1.83	35
Cecilvale	40	12	18.2 (3–35)	67	17.5 ± 0.28	10.2 ± 0.34	58
Billa Billa	34	14	11.8 (0.5–25)	73	13.6 ± 0.47	8.3 ± 0.56	61
Thallon	59	16	12.3 (2–23)	64	7.5 ± 2.18	4.4 ± 1.26	59
Riverview	18	13	7.0 (0.5–20)	14	15.7 ± 0.51	9.4 ± 0.39	60

* Range of years in parenthesis.

** Depth adjusted to “equivalent sampling depth” to account for change in soil bulk density compared to virgin situation.

**Table 5.** Changes in soil organic carbon at two sites in Chile on volcanic soils following conversion from natural grassland (adapted from Zagal et al., 2013).

Land management	SOC concentration %	SOC as percentage of that in natural grassland %
*Diguillin.* *25% clay, 41% silt. Clay contains 8% allophane* *Measurements made 8 years after clearing natural grassland* *Sampled 0–10 cm depth*		
Natural grassland	6.3	100
Continuous arable cropping (mean of 2 rotations) ^a^	4.5	71
2 yr arable, 2yr red clover (repeated)	5.0	80
2 yr arable, 2yr alfalfa (repeated)	4.5	71
3 yr arable, 5yr white clover	5.2	83
3 yr arable, 5yr alafalfa	5.0	80
		
*Santa Barbara.* *25% clay, 66% silt. Clay contains 50% allophane* *Measurements made16 years after clearing natural grassland* *Sampled 0–20 cm depth*		
Natural grassland	10.3	100
Bare soil	8.3	81
Continuous arable cropping (mean of 3 rotations) ^b^	9.6	94
3 yr subterranean clover, 1 yr winter wheat (repeated)	10.2	99
3 yr naturally regenerated pasture, 1 yr winter wheat	10.4	101

^a^ Arable crops included: sugar beet, maize, winter wheat, beans, barley.

^b^ Arable crops included: winter wheat, oats, oilseed rape, lentil.

The results from the Santa Barbara site, with high allophane content, are especially striking. Even where soil was kept bare for 16 years SOC only declined to 81% of the original value under natural grassland (Table 5). By comparison, where old grassland at Rothamsted, UK, was ploughed and kept bare SOC had decreased to about 60% of the starting value after 16 years (Barré et al., 2010). In the continuous arable treatments at Santa Barbara, SOC was still 94% of the original value after 16 years. In the long-leys (3-year leys followed by one year of winter wheat), repeated four times, there was no measurable loss of SOC. Although this is a mainly pasture rotation, with arable crops only grown 1 year in 4, it is a remarkable demonstration of the strong retention of organic matter in allophanic soils.

### Zero tillage

Several reviews comparing SOC in zero tilled and conventionally tilled soils show a small trend towards increased SOC under zero tillage in many, though not all, situations (Angers and Eriksen-Hamel, 2008; Govaerts et al., 2009; Powlson et al., 2012). The clearest effect of zero tillage is to concentrate SOC close to the soil surface, but increases in the surface 10 cm are often accompanied by decreases in deeper layers, especially around the base of the former plough depth. However, the analysis by Angers and Eriksen-Hamel (2008) shows some tendency for total SOC stock in the soil profile under zero tillage to increase over time, especially when continued for at least 15 years. These reviews are mainly based on data from temperate regions, with a strong bias towards sites in North America. Powlson et al. (2016) reviewed data from tropical or sub-tropical regions in Sub-Saharan Africa and the Indo-Gangetic Plains; SOC stock increases were somewhat larger than in temperate regions at 0.5–1 t C ha^−1^ yr^−1^, though again with considerable variation between sites. In addition, even where increases occur, it would not be expected that they would continue indefinitely. In contrast to these trends for a fairly small effect of zero tillage on SOC there is a body of evidence, mainly from Brazil, showing a marked trend for zero tillage to be effective at preserving SOC after clearance of native Cerrado vegetation growing in highly weathered oxisol soils in tropical environments. Using a chronosequence approach Corbeels et al. (2016) reported that 26 years after clearing native Cerrado vegetation in two regions in Brazil, SOC stock in the 0–40cm soil depth under a conventionally tilled maize/soybean rotation (one crop per year) had declined to 82% of that under native vegetation. But at sites where, 13 years after clearance, management had changed to zero tillage and maize/soybean double cropping (two crops per year) SOC stock had recovered to the same as that under native vegetation. The authors attributed the increase in SOC to a combination of zero tillage leading to decreased SOC decomposition and decreased soil erosion and increased organic C inputs under double cropping. It is interesting that even under conventional tillage and single cropping the SOC loss following clearance (18% after 26 years) was considerably less than at most other sites reported in this paper from elsewhere in the world. It seems likely that the high clay content of the soil (50–65%) and its acidity (pH 4.7 under native Cerrado, increasing to 5.5–5.9 under cropping as a result of liming) are key factors due to stabilization of SOM through association with aluminium oxides. One might speculate that this stabilization is magnified by decreased breakdown of aggregates under zero tillage and nitrogen inputs from biological fixation by soybean facilitating C sequestration (van Groenigen et al., 2017). Marchão et al. (2009) reported somewhat similar results at a cleared Cerrado site with an oxisol soil containing 60% clay and a pH of about 5.0 in topsoil. Fourteen years after clearance and conversion to continuous cropping, mainly a single cropping maize/soybean rotation, SOC stock in the 0–30 cm soil layer had not declined significantly compared to that under native vegetation. SOC under zero tillage was slightly greater than conventional tillage, but the difference was not significant.

The apparent stability of organic matter in the highly weathered acidic soils of the Cerrado region of Brazil is a significant exception to the more general trend of considerable and rapid SOC loss after clearance because of the large area involved: 23% of Brazil's land area and undergoing rapid clearance and conversion to agriculture (Corbeels et al., 2016). Despite the greater retention of SOC than in different environments and the possibility of an increase under zero tillage, clearance does lead to loss of the C in vegetation as this is normally burned.

## Concluding comments

With the exceptions discussed above, and noting that the situation with acidic oxisols in Brazil is a particularly significant exception, there is overwhelming evidence that it is not feasible to achieve the same content of organic C in soil under primarily arable agriculture as that under natural vegetation. Therefore, for the vast majority of situations globally, it is completely inappropriate to make this a goal for arable soils as part of a climate change mitigation strategy based on soil carbon sequestration, or any other policy objective. However, adopting management practices that either increase SOC, or limit its decline, is highly beneficial because this leads to improved soil properties and functioning that are relevant to:

- longer-term sustainability of agricultural production;
- the role of soils in the wider environment, contributing to many of the UN Sustainable Development Goals.

Improvements related to increased SOC include the following, though their magnitude and relative significance will vary with soil type, climate, location within landscapes, and agricultural system: more rapid water infiltration, reduced erosion and transport of sediments to surface waters, decreased risk of floods, improved aggregate stability and pore space and the connectivity of pores increasing the ease with which roots can proliferate, increased biological activity, and increased nutrient availability from organic matter mineralization (Johnston et al., 2009; Milne et al., 2015; Bacq-Labreuil et al., 2018; Powlson and Neal, 2021).

Farmers have recognized many of these benefits for centuries and the practices conducive to increasing organic C are well known and, in some situations, widely practiced. They include the judicious use of manures and composts, return of crop residues, avoiding long periods of bare soil through the use of cover crops, crop rotations that include periods of pasture comprising mixtures of grasses, herbs or legumes, and reduced tillage. Unfortunately, it seems that since the mid-20th century the value of these practices has been somewhat downplayed, perhaps because of the success of new crop varieties and the use of fertilizers and other technologies in increasing crop yields. In western Europe an important factor has been the decline of mixed farming systems and the tendency for arable and animal-based agriculture to become separated, mainly due to economic pressures. The benefits of reintegrating crop and livestock systems, both in Europe and globally, are discussed in detail by Schut et al. (2021) and Giller et al. (2021a).

In recent decades there has been renewed interest in practices beneficial to SOC, in part driven by interest in carbon sequestration for climate change mitigation but also for reasons of soil health and sustainability. In some regions of the world, especially Africa and South Asia, a set of practices termed conservation agriculture (CA) have been widely promoted. CA is defined as comprising three components: soil surface cover through residue retention or growing plants, reduced tillage, and crop diversification (as opposed to monocultures) (Jat, et al., 2012). Whilst there are clear barriers to full implementation of these practices, especially for resource-poor smallholder farmers (Giller et al., 2011), following at least some of the practices has a positive impact on SOC (Powlson et al., 2016) and generally, though not invariably, are likely to enhance long-term agricultural sustainability (Steward et al., 2019). The term “regenerative agriculture” has also become widely used; although the practices are less clearly defined they include many that have a positive impact on SOC (Giller et al., 2021b).

Even though the numerous benefits of increased SOC are clear, several barriers to adoption of SOC-friendly practices must be recognized. First, for individual farmers, moving to such practices often involves costs, whether directly financial or through the need for increased time and/or labour, and the need to seek new markets if the change involves diversification of crops (Schut et al. (2021). Second, in the short- or medium-term, increased SOC does not necessarily lead to increased crop yields. This was demonstrated by Hijbeek et al. (2017) who compared crop yields in treatments with and without manure (and hence high or low SOC content) in long-term experiments in Europe. When the influence of N supply from SOM was removed it was found that the direct influence of increased SOC content on yields was unexpectedly small. However, it was notable that with spring-sown crops that have a much shorter growing season than those sown in autumn there was a significant benefit to yield. This is also seen when comparing spring-sown barley with autumn-sown wheat in the Rothamsted long-term experiments (Macdonald et al., 2017, 2020). It seems likely that this results from improved soil physical structure in higher-SOC soil, permitting more rapid and extensive root growth; a factor of greater importance to short-duration crops compared to autumn-sown crops which often have a growing season of about 10 months in temperate regions, giving time to recover from periods of inhibited growth resulting from poorer soil condition. The benefit of increased SOC for shorter-duration crops will be applicable to the large areas globally where two crops are grown each year.

Although large increases in SOC are difficult to achieve in arable agriculture (Poulton et al., 2018), even small increases can have disproportionately large positive effects on various soil properties. This was seen in a review of the impacts of cereal straw retention on soil C (Powlson et al., 2011). In most cases the impact of straw retention, compared to straw removal, on total SOC was small; increases were only statistically significant in six out of 25 experiments. However, in some cases where there was virtually no measurable increase in total SOC there were substantial improvements in biological properties such as microbial biomass C content or physical characteristics such as aggregate stability and water infiltration rate.

In addition to SOC impacts on soil functioning (soil health), increases in arable soils resulting from management changes may contribute to climate change mitigation if the additional C is derived from increased photosynthetic input to soil, as for inclusion of cover crops where they were previously absent. But care is required to determine whether an increase in SOC is genuinely contributing to mitigation. For example, increases from manure applications generally *do not* contribute because the C in manure has already been transferred from atmospheric CO_2_ to the plants eaten by animals and manure is almost always utilized in some way. Thus, manure applications should be regarded as a relocation of C within the landscape rather than an additional transfer from atmosphere to land (Poulton et al., 2018; Powlson et al., 2011). The largest impact of reduced tillage is an alteration in the depth distribution of SOC, with a larger proportion being deposited near the soil surface, though in the long-term there also appears to be an overall increase due to a slowing of SOC decomposition (Powlson et al., 2012; Angers and Eriksen-Hamel, 2008). In this context we conclude that increasing SOC in agricultural soils can make some contribution to climate change mitigation, but it is necessary to ensure that the practical limitations are recognized, as opposed to the estimated theoretical potential. For example, the “4 per 1000” initiative sets an “aspirational goal” of increasing SOC in agricultural soils by 0.4% of the initial value annually as a means of counteracting anthropogenic CO_2_ emissions (Rumpel et al., 2020). However, a modelling studying based on soils in France suggests the impossibility of realizing this desirable outcome in actual practice. Martin et al. (2021) estimated that organic C inputs to soils would need to increase by 30–40% in order to achieve this rate of SOC increase: it is extremely difficult to see how this increased input could be obtained.

An overall conclusion is that it is absolutely appropriate to encourage agricultural management practices that increase SOC but, in view of the practical barriers influencing decision making by farmers, policy and/or financial measures will often be required to ensure uptake. Any increases in SOC are likely to improve soil functioning but the evidence we have reviewed shows that, in most situations, it is completely unrealistic to suggest that SOC in arable soils will reach levels comparable to those under natural vegetation. We suggest that a more appropriate strategy, for both climate change mitigation and soil health, is a twin-track approach. Within land used primarily for agriculture, aim for maximum production but within sensible constraints to minimize adverse environmental impacts and include practices that increase soil C wherever possible. Concurrently, take strong measures to minimize expansion of agriculture through deforestation, ploughing of natural grasslands or draining of peats and wetlands, thus preserving natural or semi-natural ecosystems with their large SOC stocks and numerous other ecological benefits. This requires a reversal of the trend observed over recent decades (Noon et al., 2021). Our proposed strategy is more realistic than a pretence that some notion that “natural” conditions can be reproduced in soils required for food production.
